# Latest Advances in Metasurfaces for SERS and SEIRA Sensors as Well as Photocatalysis

**DOI:** 10.3390/ijms231810592

**Published:** 2022-09-13

**Authors:** Grégory Barbillon

**Affiliations:** EPF-Ecole d’Ingénieurs, 55 Avenue du Président Wilson, 94230 Cachan, France; gregory.barbillon@epf.fr

**Keywords:** metasurfaces, SERS, SEIRA, photocatalysis, plasmonics, sensors

## Abstract

Metasurfaces can enable the confinement of electromagnetic fields on huge surfaces and zones, and they can thus be applied to biochemical sensing by using surface-enhanced Raman scattering (SERS) and surface-enhanced infrared absorption (SEIRA). Indeed, these metasurfaces have been examined for SERS and SEIRA sensing thanks to the presence of a wide density of hotspots and confined optical modes within their structures. Moreover, some metasurfaces allow an accurate enhancement of the excitation and emission processes for the SERS effect by supporting resonances at frequencies of these processes. Finally, the metasurfaces allow the enhancement of the absorption capacity of the solar light and the generation of a great number of catalytic active sites in order to more quickly produce the surface reactions. Here, we outline the latest advances in metasurfaces for SERS and SEIRA sensors as well as photocatalysis.

## 1. Introduction

During the last two decades, the using of metasurfaces has increased significantly with applications in various domains such as light nanosources [1,2], quantum devices [3,4], photovoltaics [5,6,7], photodetectors [8,9,10], planar optics [11,12,13], holography [14,15,16], lenses [17,18,19], an optical cloak [20,21,22,23], far-field thermal emission [24,25,26,27], near-field radiative energy transfer [28,29,30,31], second and third harmonic generation [32,33,34,35], refractive index sensing [36], surface-enhanced infrared absorption (SEIRA) [37,38,39,40,41], surface-enhanced Raman scattering (SERS) [42,43,44,45,46] and photocatalysis [47,48,49,50]. The metasurfaces are generally constituted of nanoresonators (building blocks) named meta-atoms whose dimensions are smaller than the incident light wavelength. Indeed, these blocks of meta-atoms can be periodically organized to realize an one-dimensional chain, a two-dimensional metasurface and a three-dimensional metamaterial. Their properties derive not from those of the materials of natural origin but come from their artificial nanostructuration, which can be controlled by adjusting their geometric parameters (organization, shape and size) [51,52]. Thus, myriad applications are workable compared to naturally arising materials. Metasurfaces have several key advantages such as the confinement of electromagnetic fields on huge surfaces, greater absorption compared to conventional materials under their bulk form, a spatially localized absorption, a tunable light absorption on a whole solar spectrum and a focusing of densities of charge-carriers in the vicinity of active reaction locations. Moreover, metasurfaces can operate like open cavity structures where light is confined by structuring the building blocks of meta-atoms [53,54]. In addition, the metasurfaces mainly used in the literature can be classified into two groups—the first one being dielectric metasurfaces and the second one being plasmonic metasurfaces. The dielectric metasurfaces are based on the Mie resonances of dielectric nanostructures which have a high refractive index [55,56,57] and for the plasmonic metasurfaces on surface plasmon resonances of metallic nanostructures [53,58,59].

In this review paper, the aim was to introduce the latest advances (2019–2022) in metasurfaces for sensing by surface-enhanced spectroscopies and for photocatalysis. In addition, several review papers on metasurfaces already exist in the scientific literature but with different focus points [11,12,13,51,53,54]. Here, we will concentrate on the metasurfaces for SERS sensors, then for SEIRA sensors, and lastly for photocatalysis.

## 2. Metasurfaces for Surface-Enhanced Raman Scattering Sensors

In this first part, the latest advances in metasurfaces for surface-enhanced Raman scattering (SERS) are introduced (see Table 1). For SERS sensors, the plasmonic or dielectric metasurfaces enable one to confine local electromagnetic fields in the vicinity of optical antennas resonating in a large spectral range matching with the excitation or Raman emission wavelengths or both, on huge domains. The SERS signal can be improved with an enhancement factor (EF) of ${\left|E\right|}^{4}$/$|E_{0}{|}^{4}$.

Sarychev et al. related the use of a metal-dielectric metasurface for the SERS detection of molecules of 4-mercaptophenylboronic acid (4-mPBA). This metasurface was constituted of a modulated dielectric coated by a silver layer, permitting the matching of plasmonic resonances to the excitation wavelength (785 nm) [60]. Chen et al. demonstrated a SERS detection of 4-aminothiophenol (4-ATP) molecules via a plasmonic metasurface consisting of a superlattice sheet of gold nanopolyhedrons (AuNPHs). An EF of $1.7\times 10^{6}$ was achieved thanks to the matching of the plasmonic resonance of this superlattice sheet of AuNPHs with the excitation wavelength of 633 nm, whose associated electric fields were strongly confined and induced by powerful interparticle coupling [61]. The group of Petti demonstrated the SERS detection of the hepatitis A virus (HAV) and 4-mercaptobenzoic acid (4-MBA) molecules by employing a metasurface constituted of gold inverted nanopyramids. An EF comprised between $5\times 10^{6}$ and $6\times 10^{6}$ was reached. This enhancement owed to a significant peak in absorbance (plasmonic resonance) near the excitation wavelength (532 nm) [62]. In addition, this same group reported the SERS detection of Shiga toxins by using a metasurface composed of gold octupolar nanostructures. The authors obtained an EF of $9\times 10^{7}$ due to the presence of a plasmonic double resonance in the visible and near-infrared regions, one of which had a plasmonic resonance close to the excitation wavelength (785 nm) used for SERS measurements [63]. Caligiuri et al. introduced the SERS detection of 1,4-benzenedithiol molecules via a plasmonic metasurface consisting of a square array of cellulose nanoholes covered by a thin layer of silver. The authors attained an enhancement factor of 6 for the SERS signal from the metasurface compared to a flat silver film. This increase in the SERS signal was due to the overlapping of the plasmonic mode corresponding to the low-energy bonding optical mode with the excitation wavelength of 785 nm [64].

**Figure 1:**
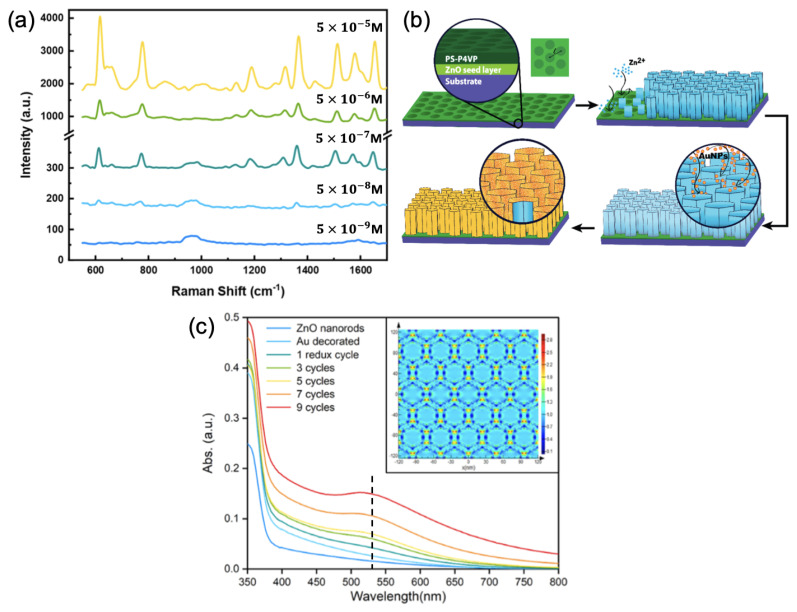
(**a**) SERS spectra of R6G molecules on the plasmonic metasurface for different R6G concentrations; (**b**) illustration of the fabrication process of the plasmonic metasurface composed of gold-coated ZnO nanorods; and (**c**) absorbance spectra of the plasmonic metasurface at different reduction cycles. The black dashed line corresponds to the excitation wavelength for SERS measurements. Inset displays the electric field mapping in the Au/ZnO nanorod array. All the figures are reprinted (adapted) with permission from [65], Copyright 2021 American Chemical Society.

Additionally, Gabinet and Osuji showed a SERS detection of rhodamine 6G (R6G) molecules through a plasmonic metasurface (see Figure 1a,b) consisting of an array of zinc oxide (ZnO) vertical nanorods (NRs) coated with a gold layer. An EF of $2\times 10^{4}$ was reached by a dint of the overlapping of a plasmonic resonance of this Au/ZnO NR array with the excitation wavelength (532 nm employed for SERS measurements, see Figure 1c), and the spatial localization (vicinity of Au/ZnO NR tops, see inset of Figure 1c) of the electric field [65]. In another way, Narasimhan et al. realized a flexible metasurface composed of a quasi-(dis)ordered group of Au nanodisks on nanoholes isolated by a silicon dioxide nanogap (in a metal–insulator–metal arrangement) for the SERS detection of human tear uric acid (UA). Enhancement factors comprised between $10^{6}$ and $5\times 10^{6}$ were achieved due to the broadband plasmonic resonance overlapping with the excitation wavelength as well as the emission wavelengths of some Raman peaks [66]. Yang et al. have fabricated a metasurface constituted of Au nanoholes hexagonally arranged on a SiO${}_{2}$ film itself deposited on a gold mirror for the SERS detection of benzenethiol molecules. The authors demonstrated by adjusting the structure parameters that a very strong absorption and a significant enhancement of electric near-fields at the wavelength used for SERS measurements were reached, thus allowing to obtain an EF of $10^{6}$ [67]. Zhao et al. employed a metasurface consisting of hyperbolic metaparticles composed of five gold layers isolated by alumina (Al${}_{2}$O${}_{3}$) layers for the SERS sensing of 4-ATP molecules. The dimensions of these hyperbolic metaparticles were tuned to obtain a scattering resonance at 785 nm, thus overlapping with the excitation wavelength employed for Raman measurements, resulting in an EF of $10^{6}$ [68]. Furthermore, Ma et al. created an electrotunable plasmonic metasurface for the SERS detection of 4-mercaptobenzoic acid (4-MBA) molecules. This metasurface consisted of Au nanoparticle arrays deposited on titanium nitride (TiN)/Ag substrate (electrode).

**Figure 2:**
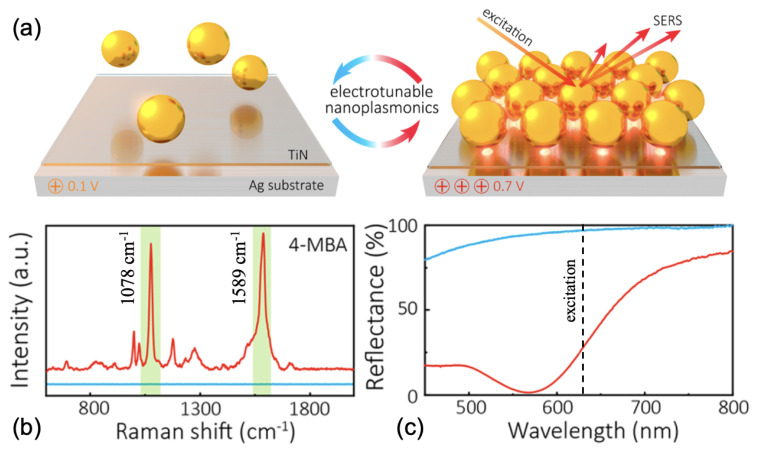
(**a**) Illustration of the electrotunable plasmonic metasurface composed of gold nanoparticles assembled on TiN/Ag substrate at 0.1 V vs. PZC (at left) and 0.7 V vs. PZC (at right). (**b**) SERS spectra of 4-MBA adsorbed on the metasurface at 0.1 V vs. PZC (blue color) and 0.7 V vs. PZC (red color). (**c**) Reflectance spectra of the plasmonic metasurface at 0.1 V vs. PZC (blue color) and 0.7 V vs. PZC (red color). The dashed black line corresponds to the excitation wavelength used for SERS measurements. All the figures are reprinted (adapted) with permission from [69], Copyright 2020 American Chemical Society.

The authors observed an EF of $10^{5}$ for a potential of 0.7 V versus potential of zero charge (PZC) where the gold nanoparticles were densely assembled (see Figure 2a,b). This EF was due to a stronger plasmonic coupling between gold nanoparticles (NPs) and the redshift of plasmonic resonance towards the excitation wavelength (633 nm; see the red curve in Figure 2c) upon the densification of these gold NPs when a potential of 0.7 V vs. PZC was applied [69]. In another way, Kovalets et al. have created a metasurface by metallic deposition (thermal sputtering of gold or silver) on track-etched membranes, then stretched leading the generation of microcracks in the metal layer for SERS sensing of malachite green (MG) molecules. The authors have noted that the number of microcracks increased when the deformation (stretching) of samples increased, thus inducing an increase in the SERS signal of MG molecules. This increase in the SERS signal is owed to the increase in the number of hotspots (zones of strong electric fields) at the level of microcracks [70]. Nguyen et al. studied the SERS sensing of R6G molecules via a metasurface consisting of a monolayer of polystyrene (NS) nanospheres coated by a thin layer of silver deposited on silver mirror. An EF of $10^{8}$ was achieved thanks to the matching of the broadband plasmonic resonance of the metasurface with the excitation wavelength as well as the emission wavelengths of Raman bands of R6G molecules [71]. Zhang’s group realized a broadband SERS substrate for the detection of R6G molecules consisting of a plasmonic metasurface composed of large silver (Ag) nanoparticles on which small aggregates of silver were obliquely deposited. Enhancement factors from $4\times 10^{7}$ to $9\times 10^{7}$ were achieved due to the matching of the broadband plasmonic resonance of the metasurface with the three excitation wavelengths using Raman measurements [72]. Wang et al. investigated a plasmonic metasurface composed of Ag nanocube (NC) arrays with an average gap between NCs of 1 nm for the detection of crystal violet (CV) molecules and several drugs such as lidocaine and methotrexate. An EF of $10^{7}$ was obtained, and this was due to the strong electric fields located in the nanogaps between Ag NCs at the excitation wavelength used for Raman experiments [73]. To finish this section dedicated to the metasurfaces for SERS sensors, the last three works concern studies based on metasurfaces using metallic nanogaps. For the first example, Luo et al. fabricated a plasmonic metasurface composed of ring-shaped nanogap arrays realized in gold film for the SERS sensing of R6G molecules. The authors obtained an EF of $3\times 10^{8}$ owing to the matching of the broad plasmonic resonance with the excitation wavelength for SERS measurements, and regions of strong electric fields located in the gap zone [74].

**Figure 3:**
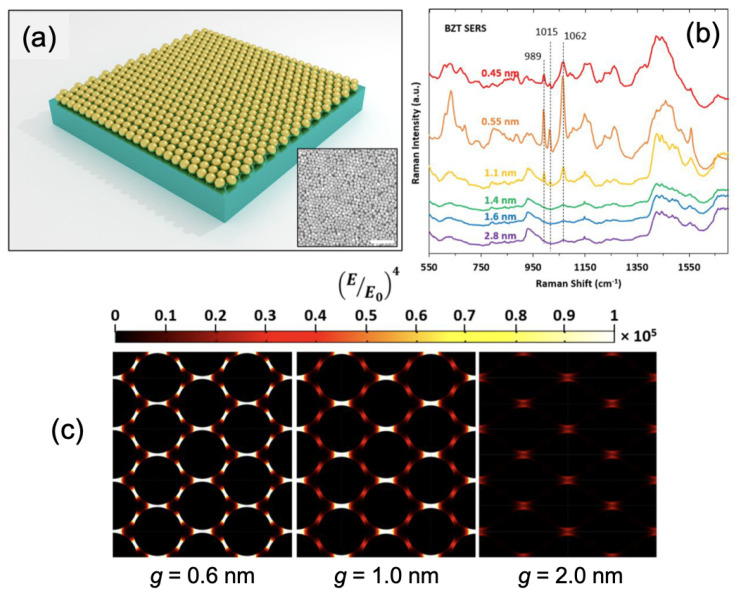
(**a**) Illustration of the plasmonic metasurface composed of gold nanospheres self-assembled on silicon substrate. Inset corresponds to a TEM picture of the plasmonic metasurface with gaps of 0.55 nm (white bar = 100 nm) (**b**) SERS spectra of benzenethiol adsorbed on the metasurface with different values of nanogap. (**c**) Mappings of the electric field enhancement for three values of nanogap (g = 0.6 nm, 1.0 nm and 2.0 nm). All the figures are reprinted (adapted) with permission from [75], Copyright 2022 American Chemical Society.

Then, Bauman et al. reported the SERS detection of benzenethiol molecules through a plasmonic metasurface composed of gold nanospheres (AuNS) with a tunable gap between AuNS (see Figure 3a,b). EFs from $10^{4}$ to $10^{5}$ were obtained thanks to the overlapping of the excitation wavelength and the wavelength of the absorbance peak of the metasurface as well as to strong electric fields obtained in the nanogaps at the excitation wavelength (see Figure 3c), for an optimal gap width of 0.55 nm [75]. Zhang et al. related the fabrication of a plasmonic metasurface consisting of an array of chiral nanogaps for the selective SERS detection of the L-cysteine and D-cysteine molecules. The authors showed that chiral nanogaps with a gap size of 5 nm were optimal for this SERS detection because their circular dichroism activity and g-factor were more important. These L-chiral nanogaps allowed obtaining the SERS spectrum of L-cysteine with more intense peaks than for the D-cysteine, and the R-chiral nanogaps allowed obtaining the SERS spectrum of D-cysteine with more intense peaks than for the L-cysteine [76]. In addition, Thareja et al. reported the use of anisotropic plasmonic metasurface composed of an array of the parallel nanogrooves fabricated into a gold film for the SERS detection of characteristic Raman peaks (G and 2D) of graphene. The SERS intensities of G and 2D Raman peaks for graphene were improved of a factor of 25–50. This enhancement of the SERS signal was owing to the excitation of propagating gap plasmons in the nanogrooves generating stronger electric fields in controlled directions [77]. To finish this first part, Jiang et al. fabricated a plasmonic metasurface composed of an array of Au-coated silica nanoflowers for the SERS sensing of benzoic acid molecules. An EF of $10^{6}$ was reached and this was due to the significant electric fields located in the gaps between nanopetals generated by the near-field coupling between these same nanopetals [78].

## 3. Metasurfaces for Surface-Enhanced Infrared Absorption Sensors

In this part, we present the latest advances in metasurfaces for surface-enhanced infrared absorption (SEIRA) (see Table 2). For this application, the plasmonic or dielectric metasurfaces allow the improvement of the infrared absorption of molecules by the confinement of local electromagnetic fields in the vicinity of optical antennas resonating in the mid-infrared (mid-IR) domain on vast areas. The SEIRA signal will increase with an enhancement factor (EF) of ${\left|E\right|}^{2}$/$|E_{0}{|}^{2}$, when the frequency matching between plasmonic or dielectric nanostructures and molecular vibration modes occurs.

**Figure 4:**
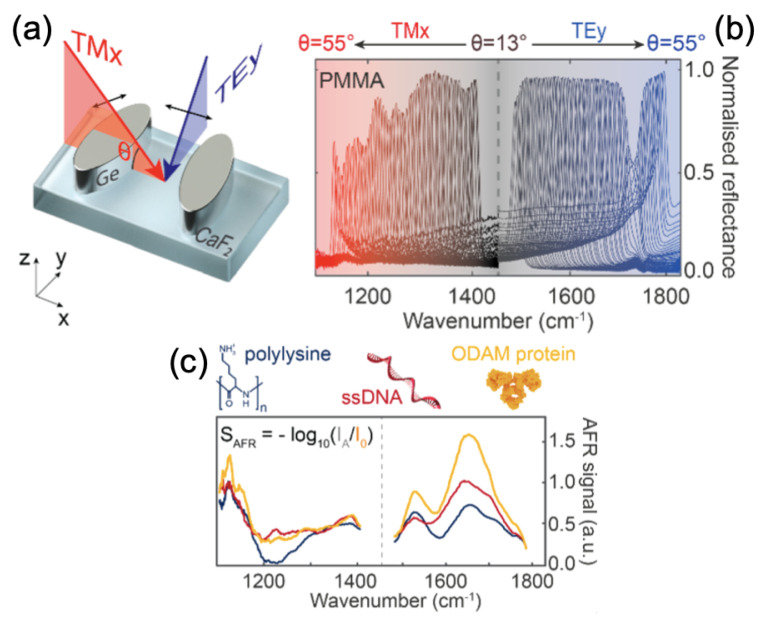
(**a**) Principle scheme of the unit cell for the dielectric metasurface. (**b**) Normalized reflectance spectra for the different incidence angles of light. (**c**) Absorbance spectra of polylysine, ssDNA and ODAM. All the figures are reprinted (adapted) with permission from [80], Copyright 2021 American Chemical Society.

As first example, Altug’s group showed the detection of human odontogenic ameloblast-associated protein (ODAM), polylysine and single-stranded DNA (ssDNA) by using dielectric metasurfaces with high-quality factors. The resonance for this dielectric metasurface was controlled with the incidence angle of light and the polarization in order to obtain molecular fingerprint details. In addition, the building block (meta-atom) of this dielectric metasurface was composed of two elliptical shapes in germanium realized on a calcium fluoride (CaF${}_{2}$) substrate (see Figure 4a), and the principal axes of two ellipses were rotated asymmetrically between them. This metasurface thus permitted enhancing the electromagnetic near-fields (EF = 6000) located at the tips of elliptical shapes, where the interactions between biomolecules and light occur. Finally, by employing this approach, the detection of mid-IR absorption fingerprints of biomolecules (here: polylysine, ssDNA, and ODAM, see Figure 4b,c) was realized without any tunable laser and/or expensive spectrometers that take up space [79,80]. Another example of this same research group is presented. In this example, the authors used pixelated dielectric metasurfaces for the detection of proteins A/G by SEIRA. Each metapixel of this metasurface was constituted of an array of anisotropic hydrogenated amorphous silicon ellipses (with an EF = 1500 and the same geometry as the previous example, as can be seen in Figure 4a), whose resonance can be set over a given range of fingerprints of target molecules by adjusting the lateral dimensions of the unit cell by a factor named *S*. Thus, thanks to this pixelated dielectric metasurface, they accessed the signals of biomolecules over an expanded spectrum of wavelength with a great sensitivity [80].

**Figure 5:**
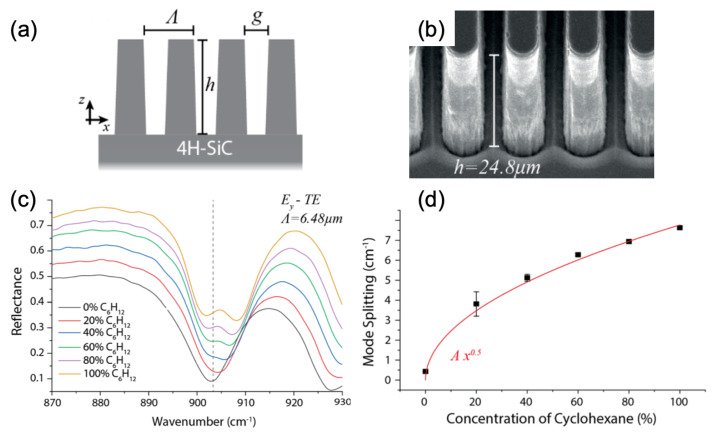
(**a**) Principle scheme of 4H-SiC high-aspect-ratio gratings (HAGs) with the tooth height *h* of grating, the grating period Λ and the tooth spacing of grating *g*. (**b**) SEM image of the 4H-SiC HAG. (**c**) Reflectance spectra with different concentrations of cyclohexane. The dashed line corresponds to the vibration mode of cyclohexane located at 903 cm${}^{-1}$. (**d**) Mode splitting versus cyclohexane concentration. The red line corresponds to the fit with a square root function. All the figures are reprinted (adapted) with permission from [81], Copyright 2020 American Chemical Society.

Folland et al. demonstrated the sensing of cyclohexane using a metasurface composed of 4H-silicium carbide (4H-SiC) high-aspect-ratio gratings (HAGs, see Figure 5a,b). Thanks to this metasurface, a vibrational coherent coupling between the epsilon-near-zero (ENZ) waveguide mode and a vibrational mode of cyclohexane was evidenced by the mode splitting coming from the interaction between these two modes and following a dependence in $\sqrt{C}$ with the cyclohexane concentration (see Figure 5c,d) [81]. The work of Leitis et al. has related the use of plasmonic metasurfaces in order to observe the liposome catching. These plasmonic metasurfaces were fabricated on alumina (Al${}_{2}$O${}_{3}$) membranes and they were composed of aluminum (Al) nanoantennas (or meta-atoms), thus enabling a conception with various resonances in the mid-IR. Thereby, these resonances enhanced and overlapped the absorption bands of the liposomes in this case. To resume, the authors succeeded in demonstrating the catching of liposomes by SEIRA thanks to the high electric field enhancements (EF = 30–160) located at the hotspots of Al nanoantennas whose resonances overlapped with the absorption bands of the liposomes [82]. To finish this section, Autore et al. reported the sensing of 4,4${}^{\prime }$-bis(N-carbazolyl)-1,1${}^{\prime }$-biphenyl (CBP) molecules using a metasurface composed of monoisotopic hexagonal boron nitride (h-BN) ribbon arrays realized on a CaF${}_{2}$ substrate. They observed a SEIRA signal increase of a factor of 10 for CBP molecules with this monoisotopic h-BN metasurface compared to the reference sample without the metasurface. This increase in the SEIRA signal was due to coupling between hyperbolic phonon-polariton modes and vibration modes of CBP molecules [83].

## 4. Metasurfaces for Photocatalysis

In this last part, we introduce the latest advances in metasurfaces for photocatalysis (see Table 3). For photocatalysis, a broadband absorption of light and an enhancement of photocurrents are required. Thus, by adjusting the size, shape and organization of building blocks, the plasmonic or dielectric metasurfaces will improve the photon–electron energy conversion, thus enabling the enhancement of this absorption capacity of the solar light and the generation of a great number of catalytic active sites in order to more quickly produce the surface reactions. In addition, the enhancement factor is defined for the following examples as the ratio of photocurrents or reaction rates obtained for the metasurface and the reference sample (metasurface/reference).

At first, Ozbay’s group demonstrated a catalyzed reaction of the hydrogen evolution (HER) using a metasurface constituted of random CrO${}_{x}$–NiO${}_{x}$ nanorods. CrO${}_{x}$ nanorods were chosen for their quality of light absorption in zones close to the surface thanks to hotspots generated by this geometry of random nanorods, and for easing the separation of charges. NiO${}_{x}$ was chosen as an HER catalyst. For the CrO${}_{x}$–NiO${}_{x}$ metasurface (CrO${}_{x}$ metasurface covered by a NiO${}_{x}$ layer, and then annealed at 600 °C over 30 min), the authors have shown an increase in the photocurrent values of a factor of 30 and 3 compared to those of a planar conception and those of a bare CrO${}_{x}$-30 metasurface (30 = annealing at 600 °C over 30 min), respectively. This increase was due to the separation of charges which triggered the HER. This separation of charges was possible thanks to a good band alignment between the conduction bands of CrO${}_{x}$, NiO${}_{x}$ and the HER potential. In addition to the increase in photocurrent values, the ICPE measurements exhibited an enhancement of the efficiency in the conversion process [84]. Furthermore, based on similar principles, the group of Cortes also reported an increase in the photocurrent values of a factor of 5.7 compared to those of a planar conception upon HER conditions by using a different metasurface composed of amorphous gallium phosphide (a-GaP) nanodisks deposited on an indium tin oxide film itself deposited on a SiO${}_{2}$ substrate [85].

**Figure 6:**
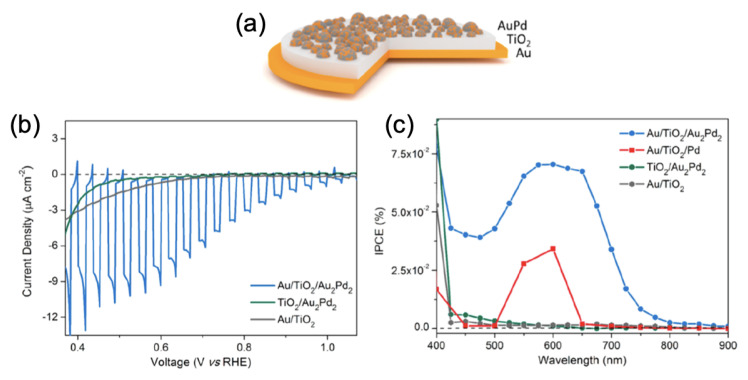
(**a**) Principle scheme of the plasmonic metasurface. (**b**) Linear sweep voltammogram scans with chopped visible light. (**c**) Incident photon conversion efficiency (IPCE) versus wavelength. All the figures are reprinted (adapted) with permission from [86], Copyright 2021 American Chemical Society.

Building upon the above, Xiao et al. reported an increase in the photocurrent values of a factor 5–20 upon the reduction of water by using a plasmonic metasurface composed of AuPd nanoparticles deposited on a bilayer film of titanium dioxide and gold itself deposited on a glass substrate (see Figure 6a,b and blue curve in Figure 6b). This increase in photocurrent was due to the mixed photon-to-energy conversion of gold and palladium through plasmonic resonances for gold and interband transitions of Pd. Moreover, the incident photon conversion efficiency (IPCE) exposed an efficiency improvement in the conversion process (see blue curve in Figure 6c) [86].

**Figure 7:**
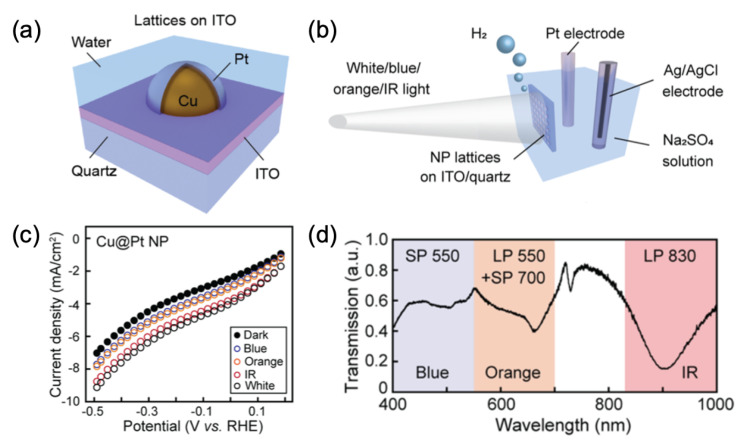
(**a**) Principle scheme of the unit cell of the metasurface. (**b**) Illustration of HER measurements. (**c**) Linear sweep voltammogram scans of HER at different wavelengths (see the caption in the curves). (**d**) Transmission spectrum of the Cu@Pt nanoparticle array, where three ranges of light are indicated by the shading of each. All the figures are reprinted (adapted) with permission from [87], Copyright 2021 American Chemical Society.

Finally, Deng et al. have shown an improvement in photocurrent of a factor of 5–20 during the HER by employing a metasurface constituted of lattices of copper–platinum core–shell nanoparticles deposited on an ITO/quartz substrate (see Figure 7a–c). This best improvement in photocurrent is owed to the surface lattice resonances of lattices of Cu@Pt nanoparticles inducing robuster absorption of light (see the IR part of Figure 7d, where the dip indicates the presence of a surface lattice resonance) and electromagnetic fields [87]. A work of Kherani’s group has reported on the enhancement of the rate of methanol production of a factor 93–181 via the use of a metasurface constituted of ZnO/Cu nanocubes on an Au/Cu bilayer film. This enhancement was obtained by combining the broadband absorption and the plasmonics, thus inducing strong electric fields over the (nonplasmonic) absorption range [88]. Capitolis et al. demonstrated an enhancement of a factor 5.7 on the reaction rate of the NO oxidation through the use of a metasurface formed of nanohole arrays realized in a silicon nitride (SiN${}_{x}$) film, then covered by a TiO${}_{2}$ layer. This enhancement was due to the significant absorption of the incident light (UV-light in this case) via slow light modes produced by the periodic lattice of metasurface [89]. Additionally, Hu et al. created a metasurface composed of loss-engineered substoichiometric titanium oxide (TiO${}_{2-x}$) ellipses and based on the bound states in the continuum (BIC) for increasing the reaction rate of silver (Ag) reduction. The authors have reported an EF of 7 for the reaction rate of Ag reduction with the low-defect TiO${}_{2-x}$ (named TiO${}_{2}$-L), which was due to the matching of the excitation wavelength with the quasi-BIC resonance of the metasurface by adjusting all the geometric parameters of the unit cell of metasurface. This matching enabled an optimal absorption of the visible light and an optimal enhancement of near-fields, thus inducing a better reaction rate [90].

**Figure 8:**
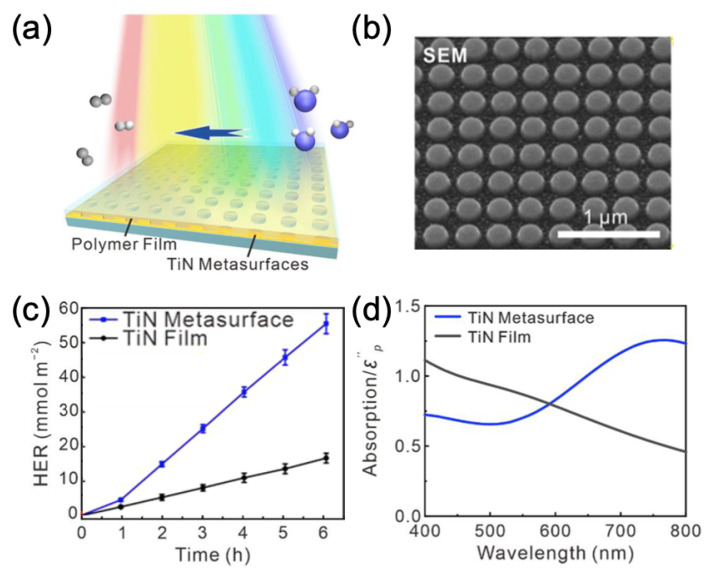
(**a**) Principle scheme of the hydrogen evolution induced by visible light by employing the TiN metasurface covered by the photocatalyst polymer. (**b**) SEM picture of the metasurface composed of TiN nanodisks. (**c**) HER rate of the photocatalyst polymer deposited on a TiN metasurface and TiN film (reference) versus time. (**d**) Calculated absorption spectra of the photocatalyst polymer deposited on the TiN metasurface and TiN film (reference). All the figures are reprinted (adapted) with permission from [91], Copyright 2021 American Chemical Society.

Yu et al. reported an improvement of a factor 3.2 for the hydrogen evolution rate by employing a metasurface composed of titanium nitride (TiN) nanodisks fabricated on a thin TiN film and arranged in square array, then covered by a photocatalyst polymer (see Figure 8a–c; TiN film without any nanodisk serves as reference). This improvement of the HER was obtained thanks to the broadband plasmon resonance of the metasurface (see Figure 8d) producing strong electric fields and thus increasing different rates, such as those of absorption light, carrier separation and the transfer of hot carriers in photocatalyst polymers [91]. To terminate this part on metasurfaces for photocatalysis, Dutta et al. reported the use of a plasmonic metasurface for the water splitting. This metasurface consisted of arrays of gold nanodisks deposited on a hematite film—itself deposited on gold film. Enhancement factors of 2 and 6 in the photocurrent of the water oxidation were obtained above and below the bandgap of hematite, respectively. The EF of 2 in the photocurrent obtained above the bandgap was owed to an improved scattering by the Au nanodisks and a back-reflection from the Au mirror. The EF of 6 in the photocurrent obtained below the bandgap was due to the hot electrons produced by plasmon decay [92].

## 5. Conclusions and Outlook

In summary, we depicted the latest advances in the performance of plasmonic or dielectric metasurfaces for SERS and SEIRA sensors as well as photocatalysis. Thanks to the performances of these metasurfaces, enhancement factors were obtained ranging from 2 to 81 for photocatalysis, from 10 to 6000 for SEIRA sensors and from $10^{4}$ to $3\times 10^{8}$ for SERS sensors. These enhancement factors were due to the confinement of local electromagnetic fields in the vicinity of optical antennas that had resonances in a large spectral range being thus able to increase the SERS and SEIRA signals. Furthermore, some metasurfaces enabled an accurate enhancement of the excitation and emission processes for the SERS effect by supporting resonances at frequencies of these processes. Another advantage of using metasurfaces is to achieve the good reproducibility and uniformity of the SERS signal on the whole substrate thanks to the present fabrication techniques, which permit the identical realization of plasmonic or dielectric nanostructures. Finally, these metasurfaces permitted the enhancement of the absorption capacity of the solar light and the generation of a great number of catalytic active sites in order to more quickly produce the surface reactions. A couple of interesting perspectives concerning the metasurfaces to be used would be chemo-tunable metasurfaces that can allow a chemical modulation to control their optical properties alternately to electrotunable metasurfaces (presented here in one or two examples), or surface-functionalized metasurfaces for handling their properties. For instance, proteins can be functionalized on metasurfaces in order to obtain very selective biochemical interactions for improving the sensing of antibodies or antigens. Finally, we think that, in the near future, chemo-tunable or surface-functionalized metasurfaces will offer perspectives for photocatalysis and sensing based on surface-enhanced spectroscopies.

## Figures and Tables

**Table 1:** Unit cell of metasurfaces, detected molecules and the enhancement factor (EF) for surface-enhanced Raman scattering sensors (4-mPBA = 4-mercaptoPhenylBoronic Acid; AuNPHs = gold NanoPolyHedrons; 4-ATP = 4-AminoThiophenol; 4-MBA = 4-MercaptoBenzoic Acid; HAV = Hepatitis A Virus; ZnO = Zinc Oxide; MIM NS = Metal–Insulator–Metal NanoStructures; Al${}_{2}$O${}_{3}$ = Alumina; MG = Malachite Green; PS = PolyStyrene; CV = Crystal Violet).

Unit Cell of Metasurfaces	Detected Molecules	EF	References
One Ag nanohole	4-mPBA	–	[60]
Six AuNPHs	4-ATP	$1.7\times 10^{6}$	[61]
Three Au pyramidal nanoholes	4-MBA, HAV	$5\times 10^{6}$–$6\times 10^{6}$	[62]
Three Au triangular nanocavities	4-MBA, Shiga Toxins	$9\times 10^{7}$	[63]
One Ag cellulose disk–hole matrix	1,4-benzenedithiol	–	[64]
Seven vertical Au/ZnO nanorods	Rhodamine 6G	$2\times 10^{4}$	[65]
Quasi-(dis)ordered MIM NS	Human tear uric acid	$10^{6}$–$5\times 10^{6}$	[66]
Seven Au nanoholes	Benzenethiol	$10^{6}$	[67]
Truncated Au/Al${}_{2}$O${}_{3}$ cones	4-ATP	$10^{6}$	[68]
Au nanoparticle assembly	4-MBA	$10^{5}$	[69]
Au/Ag microcracks	MG	–	[70]
Ag-coated PS microspheres	Rhodamine 6G	$10^{8}$	[71]
Ag aggregates on one Ag NP	Rhodamine 6G	$4\times 10^{7}$–$9\times 10^{7}$	[72]
Ag nanocube superlattice	CV, drugs	$10^{7}$	[73]
Seven ring-shaped nanogaps	Rhodamine 6G	$3\times 10^{8}$	[74]
Seven Au nanospheres	Benzenethiol	$10^{4}$–$10^{5}$	[75]
Seven Au chiral nanogaps	L/D-cysteine	–	[76]
Au nanogrooves	Graphene	–	[77]
Au-coated silica nanoflowers	Benzoic acid	$10^{6}$	[78]

**Table 2:** Unit cell of metasurfaces, detected molecules and the enhancement factor (EF) for surface-enhanced infrared absorption sensors (ssDNA = single-stranded DNA; ODAM = human odontogenic ameloblast-associated protein; a-Si:H = anisotropic hydrogenated amorphous silicon; 4H-SiC = 4H-silicium carbide; h-BN = hexagonal boron nitride; CBP = 4,4${}^{\prime }$-bis(N-carbazolyl)-1,1${}^{\prime }$-biphenyl).

Unit Cell of Metasurfaces	Detected Molecules	EF	References
Two germanium ellipses	Polylysine, ssDNA, ODAM	6000	[79,80]
Two a-Si:H ellipses	Proteins A/G	1500	[80]
Two 4H-SiC teeth	Cyclohexane	–	[81]
Five aluminum ellipses	Liposomes	30–160	[82]
Two monoisotopic h-BN ribbons	CBP	10	[83]

**Table 3:** Unit cell of metasurfaces, reactions of photocatalysis and their EF (CrO${}_{x}$ = *p*-type Chromium Oxide; NiO${}_{x}$ = *p*-type Nickel Oxide; NRs = Nanorods; PEC = Photoelectrochemical; a-GaP = amorphous Gallium Phosphide; AuPd = Gold/Palladium; NPs = Nanoparticles; Cu@Pt = Copper–Platinum Core–Shell; Zn/Cu = Zinc/Copper; TiO${}_{2}$ = Titanium Dioxide; NO = Nitrogen Monoxide; TiO${}_{2-x}$ = Loss-engineered Substoichiometric Titanium Oxide; TiN = Titanium Nitride; HM = Hematite).

Unit Cell of Metasurfaces	Photocatalysis	EF	References
Random CrO${}_{x}$–NiO${}_{x}$ NRs	PEC hydrogen generation	30	[84]
One a-GaP nanodisk	PEC hydrogen generation	5.7	[85]
Random AuPd NPs	PEC reduction of water	5–20	[86]
One Cu@Pt NP	PEC hydrogen generation	5–20	[87]
One Zn/Cu nanocube	Methanol production	93–181	[88]
One TiO${}_{2}$ nanohole	NO oxidation reaction rate	5.7	[89]
Two TiO${}_{2-x}$ ellipses	Ag reduction reaction	7	[90]
One TiN nanodisk	Hydrogen evolution rate	3.2	[91]
Au nanodisk on HM/Au bilayer	Water splitting	2–6	[92]

## Data Availability

Not applicable.

## References

[1] Wang D., Wang W., Knudson M.P., Schatz G.C., Odom T.W. (2018). Structural Engineering in Plasmon Nanolasers. Chem. Rev..

[2] Staude I., Pertsch T., Kivshar Y.S. (2019). All-Dielectric Resonant Meta-Optics Lightens up. ACS Photonics.

[3] Wang K., Titchener J.G., Kruk S.S., Xu L., Chung H.-P., Parry M., Kravchenko I.I., Chen Y.-H., Solntsev A.S., Kivshar Y.S. (2018). Quantum metasurface for multiphoton interference and state reconstruction. Science.

[4] Li L., Liu Z., Ren X., Wang S., Su V.-C., Chen M.-K., Chu C.H., Kuo H.Y., Liu B., Zang W. (2020). Metalens-array–based high-dimensional and multiphoton quantum source. Science.

[5] Ou Q.-D., Xie H.-J., Chen J.-D., Zhou L., Tang J.-X. (2016). Enhanced light harvesting in flexible polymer solar cells: Synergistic simulation of a plasmonic meta-mirror and a transparent silver mesowire electrode. J. Mater. Chem. A.

[6] Voroshilov P.M., Ovchinnikov V., Papadimitratos A., Zakhidov A.A., Simovski C.R. (2018). Light Trapping Enhancement by Silver Nanoantennas in Organic Solar Cells. ACS Photonics.

[7] Chang C.-C., Kort-Kamp W.J.M., Nogan J., Luk T.S., Azad A.K., Taylor A.J., Dalvit D.A.R., Sykora M., Chen H.-T. (2018). High-Temperature Refractory Metasurfaces for Solar Thermophotovoltaic Energy Harvesting. Nano Lett..

[8] Fang J., Wang D., DeVault C.T., Chung T.-F., Chen Y.P., Boltasseva A., Shalaev V.M., Kildishev A.V. (2017). Enhanced Graphene Photodetector with Fractal Metasurface. Nano Lett..

[9] Li L., Wang J., Kang L., Liu W., Yu L., Zheng B., Brongersma M.L., Werner D.H., Lan S., Shi Y. (2020). Monolithic Full-Stokes Near-Infrared Polarimetry with Chiral Plasmonic Metasurface Integrated Graphene–Silicon Photodetector. ACS Nano.

[10] Sharma N., Bar-David J., Mazurki N., Levy U. (2020). Metasurfaces for Enhancing Light Absorption in Thermoelectric Photodetectors. ACS Photonics.

[11] Yu N., Capasso F. (2014). Flat optics with designer metasurfaces. Nat. Mater..

[12] Genevet P., Capasso F., Aieta F., Khorasaninejad M., Devlin R. (2017). Recent Advances in Planar Optics: From Plasmonic to Dielectric Metasurfaces. Optica.

[13] Ding F., Pors A., Bozhevolnyi S.I. (2018). Gradient Metasurfaces: A Review of Fundamentals and Applications. Rep. Prog. Phys..

[14] Dolev I., Epstein I., Arie A. (2012). Surface-Plasmon Holographic Beam Shaping. Phys. Rev. Lett..

[15] Zheng G.X., Mühlenbernd H., Kenney M., Li G.X., Zentgraf T., Zhang S. (2015). Metasurface holograms reaching 80% efficiency. Nat. Nanotechnol..

[16] Deng Z.L., Jin M.K., Ye X., Wang S., Shi T., Deng J.H., Mao N.B., Cao Y.Y., Guan B.O., Alu A. (2020). Full-Color Complex-Amplitude Vectorial Holograms Based on Multi-Freedom Metasurfaces. Adv. Funct. Mater..

[17] Aieta F., Genevet P., Kats M.A., Yu N., Blanchard R., Gaburro Z., Capasso F. (2012). Aberration-Free Ultrathin Flat Lenses and Axicons at Telecom Wavelengths Based on Plasmonic Metasurfaces. Nano Lett..

[18] Pors A., Nielsen M.G., Eriksen R.L., Bozhevolnyi S.I. (2013). Broadband Focusing Flat Mirrors Based on Plasmonic Gradient Metasurfaces. Nano Lett..

[19] Ni X.J., Ishii S., Kildishev A.V., Shalaev V.M. (2013). Ultra-thin, planar, Babinet-inverted plasmonic metalenses. Light Sci. Appl..

[20] Valentine J., Li J.S., Zentgraf T., Bartal G., Zhang X. (2009). An optical cloak made of dielectrics. Nat. Mater..

[21] Gharghi M., Gladden C., Zentgraf T., Liu Y.M., Yin X.B., Valentine J., Zhang X. (2011). A Carpet Cloak for Visible Light. Nano Lett..

[22] Chen H.S., Zheng B., Shen L., Wang H.P., Zhang X.M., Zheludev N.I., Zhang B.L. (2013). Ray-optics cloaking devices for large objects in incoherent natural light. Nat. Commun..

[23] Hu J., Bandyopadhyay S., Liu Y.-H., Shao L.-Y. (2021). A Review on Metasurface: From Principle to Smart Metadevices. Front. Phys..

[24] De Zoysa M., Asano T., Mochizuki K., Oskooi A., Inoue T. (2012). Conversion of Broadband to Narrowband Thermal Emission Through Energy Recycling. Nat. Photonics.

[25] Barbillon G., Sakat E., Hugonin J.-P., Biehs S.-A., Ben-Abdallah P. (2017). True thermal antenna with hyperbolic metamaterials. Opt. Express.

[26] Xie X., Li X., Pu M., Ma X., Liu K., Guo Y., Luo X. (2018). Plasmonic Metasurfaces for Simultaneous Thermal Infrared Invisibility and Holographic Illusion. Adv. Funct. Mater..

[27] Zhang X., Zhang Z., Wang Q., Zhu S., Liu H. (2019). Controlling Thermal Emission by Parity-Symmetric Fano Resonance of Optical Absorbers in Metasurfaces. ACS Photonics.

[28] Joulain K., Drevillon J., Ben-Abdallah P. (2010). Noncontact heat transfer between two metamaterials. Phys. Rev. B.

[29] Liu B., Shen S. (2013). Broadband near-field radiative thermal emitter/absorber based on hyperbolic metamaterials: Direct numerical simulation by the Wiener chaos expansion method. Phys. Rev. B.

[30] Liu X., Zhang Z. (2015). Near-Field Thermal Radiation between Metasurfaces. ACS Photonics.

[31] Fernandez-Hurtado V., Garcia-Vidal F.J., Fan S., Cuevas E.C. (2017). Enhancing Near-Field Radiative Heat Transfer with Si-based Metasurfaces. Phys. Rev. Lett..

[32] Lee J., Nookala N., Gomez-Diaz J.S., Tymchenko M., Demmerle F., Boehm G., Amann M.-C., Alu A., Belkin M.A. (2016). Ultrathin Second-Harmonic Metasurfaces with Record-High Nonlinear Optical Responses. Adv. Opt. Mater..

[33] Fedotova A., Younesi M., Sautter J., Vaskin A., Löchner F.J.F., Steinert M., Geiss R., Pertsch T., Staude I., Setzpfandt F. (2020). Second-Harmonic Generation in Resonant Nonlinear Metasurfaces Based on Lithium Niobate. Nano Lett..

[34] Yang Y., Wang W., Boulesbaa A., Kravchenko I.I., Briggs D.P., Puretzky A., Geohegan D., Valentine J. (2015). Nonlinear Fano-Resonant Dielectric Metasurfaces. Nano Lett..

[35] Shorokhov A.S., Melik-Gaykazyan E.V., Smirnova D.A., Hopkins B., Chong K.E., Choi D.-Y., Shcherbakov M.R., Miroshnichenko A.E., Neshev D.N., Fedyanin A.A. (2016). Multifold Enhancement of Third-Harmonic Generation in Dielectric Nanoparticles Driven by Magnetic Fano Resonances. Nano Lett..

[36] Qin J., Jiang S., Wang Z., Cheng X., Li B., Shi Y., Tsai D.P., Liu A.Q., Huang W., Zhu W. (2022). Metasurface Micro/Nano-Optical Sensors: Principles and Applications. ACS Nano.

[37] Wu C., Khanikaev A.B., Adato R., Arju N., Yanik A.A., Altug H., Shvets G. (2012). Fano-resonant asymmetric metamaterials for ultrasensitive spectroscopy and identification of molecular monolayers. Nat. Mater..

[38] Brown L.V., Yang X., Zhao K., Zheng B.Y., Nordlander P., Halas N.J. (2015). Fan-Shaped Gold Nanoantennas above Reflective Substrates for Surface-Enhanced Infrared Absorption (SEIRA). Nano Lett..

[39] Chen X., Ciraci C., Smith D.R., Oh S.-H. (2015). Nanogap-Enhanced Infrared Spectroscopy with Template-Stripped Wafer-Scale Arrays of Buried Plasmonic Cavities. Nano Lett..

[40] Chae J., Lahiri B., Centrone A. (2016). Engineering Near-Field SEIRA Enhancements in Plasmonic Resonators. ACS Photonics.

[41] Neubrech F., Huck C., Weber K., Pucci A., Giessen H. (2017). Surface-Enhanced Infrared Spectroscopy Using Resonant Nanoantennas. Chem. Rev..

[42] Zhang N., Liu K., Liu Z., Song H., Zeng X., Ji D., Cheney A., Jiang S., Gan Q. (2015). Ultrabroadband Metasurface for Efficient Light Trapping and Localization: A Universal Surface-Enhanced Raman Spectroscopy Substrate for “All” Excitation Wavelengths. Adv. Mater. Interfaces.

[43] Zhang X., Zheng Y., Liu X., Lu W., Dai J., Lei D.Y., MacFarlane D.R. (2015). Hierarchical Porous Plasmonic Metamaterials for Reproducible Ultrasensitive Surface-Enhanced Raman Spectroscopy. Adv. Mater..

[44] Yang Y., Lee Y.H., Phang I.Y., Jiang R., Sim H.Y.F., Wang J., Ling X.Y. (2016). A Chemical Approach To Break the Planar Configuration of Ag Nanocubes into Tunable Two-Dimensional Metasurfaces. Nano Lett..

[45] Gwo S., Wang C.-Y., Chen H.-Y., Lin M.-H., Sun L., Li X., Chen W.-L., Chang Y.-M., Ahn H. (2016). Plasmonic Metasurfaces for Nonlinear Optics and Quantitative SERS. ACS Photonics.

[46] Abdulhalim I. (2018). Coupling configurations between extended surface electromagnetic waves and localized surface plasmons for ultrahigh field enhancement. Nanophotonics.

[47] Gao H., Liu C., Jeong H.E., Yang P. (2012). Plasmon-Enhanced Photocatalytic Activity of Iron Oxide on Gold Nanopillars. ACS Nano.

[48] Li J., Cushing S.K., Zheng P., Meng F., Chu D., Wu N. (2013). Plasmon-induced photonic and energy-transfer enhancement of solar water splitting by a hematite nanorod array. Nat. Commun..

[49] Clavero C. (2014). Plasmon-induced hot-electron generation at nanoparticle/metal-oxide interfaces for photovoltaic and photocatalytic devices. Nat. Photonics.

[50] Aslam U., Chavez S., Linic S. (2017). Controlling energy flow in multimetallic nanostructures for plasmonic catalysis. Nat. Nanotechnol..

[51] Kildishev A.V., Boltasseva A., Shalaev V.M. (2013). Planar Photonics with Metasurfaces. Science.

[52] Monticone F., Alu A. (2017). Metamaterial, plasmonic and nanophotonic devices. Rep. Prog. Phys..

[53] Meinzer N., Barnes W.L., Hooper I.R. (2014). Plasmonic meta-atoms and metasurfaces. Nat. Photonics.

[54] Vaskin A., Kolkowski R., Femius Koenderink A., Staude I. (2019). Light-emitting metasurfaces. Nanophotonics.

[55] Tittl A., Leitis A., Liu M., Yesilkoy F., Choi D.-Y., Neshev D.N., Kivshar Y.S., Altug H. (2018). Imaging-based molecular barcoding with pixelated dielectric metasurfaces. Science.

[56] Romano S., Zito G., Manago S., Calafiore G., Penzo E., Cabrini S., De Luca A.C., Mocella V. (2018). Surface-Enhanced Raman and Fluorescence Spectroscopy with an All-Dielectric Metasurface. J. Phys. Chem. C.

[57] Koshelev K., Kivshar Y. (2021). Dielectric Resonant Metaphotonics. ACS Photonics.

[58] Shi X., Ueno K., Oshikiri T., Sun Q., Sasaki K., Misawa H. (2018). Enhanced water splitting under modal strong coupling conditions. Nat. Nanotechnol..

[59] Gao J., Zhang N., Ji D., Song H., Liu Y., Zhou L., Sun Z., Jornet J.M., Thompson A.C., Collins R.L. (2018). Superabsorbing Metasurfaces with Hybrid Ag—Au Nanostructures for Surface-Enhanced Raman Spectroscopy Sensing of Drugs and Chemicals. Small Methods.

[60] Sarychev A.K., Ivanov A., Lagarkov A.N., Ryzhikov I., Afanasev K., Bykov I., Barbillon G., Bakholdin N., Mikhaillov M., Smyk A. (2022). Plasmon Localization and Giant Fields in an Open-Resonator Metasurface for Surface-Enhanced-Raman-Scattering Sensors. Phys. Rev. Appl..

[61] Chen Y., Yin H., Sikdar D., Yan L., Zhou Z., Sun J., Wang C. (2021). Gold Nanopolyhedron-Based Superlattice Sheets as Flexible Surface-Enhanced Raman Scattering Sensors for Detection of 4-Aminothiophenol. ACS Appl. Nano Mater..

[62] Palermo G., Rippa M., Conti Y., Vestri A., Castagna R., Fusco G., Suffredini E., Zhou J., Zyss J., De Luca A. (2021). Plasmonic Metasurfaces Based on Pyramidal Nanoholes for High- Efficiency SERS Biosensing. ACS Appl. Mater. Interfaces.

[63] Rippa M., Sagnelli D., Vestri A., Marchesano V., Munari B., Carnicelli D., Varrone E., Brigotti M., Tozzoli R., Montalbano M. (2022). Plasmonic Metasurfaces for Specific SERS Detection of Shiga Toxins. ACS Appl. Mater. Interfaces.

[64] Caligiuri V., Tedeschi G., Palei M., Miscuglio M., Martin-Garcia B., Guzman-Puyol S., Hedayati M.K., Kristensen A., Athanassiou A., Cingolani R. (2020). Biodegradable and Insoluble Cellulose Photonic Crystals and Metasurfaces. ACS Nano.

[65] Gabinet U.R., Osuji C.O. (2021). Plasmonic Sensing from Vertical Au-Coated ZnO Nanorod Arrays Templated by Block Copolymers. ACS Appl. Nano Mater..

[66] Narasimhan V., Siddique R.H., Park H., Choo H. (2020). Bioinspired Disordered Flexible Metasurfaces for Human Tear Analysis Using Broadband Surface-Enhanced Raman Scattering. ACS Omega.

[67] Yang K., Wang J., Yao X., Lyu D., Zhu J., Yang Z., Liu B., Ren B. (2021). Large-Area Plasmonic Metamaterial with Thickness-Dependent Absorption. Adv. Optical Mater..

[68] Zhao Y., Hubarevich A., Iarossi M., Borzda T., Tantussi F., Huang J.-A., De Angelis F. (2021). Hyperbolic Nanoparticles on Substrate with Separate Optical Scattering and Absorption Resonances: A Dual Function Platform for SERS and Thermoplasmonics. Adv. Optical Mater..

[69] Ma Y., Sikdar D., Fedosyuk A., Velleman L., Klemme D.J., Oh S.-H., Kucernak A.R.J., Kornyshev A.A., Edel J.B. (2020). Electrotunable Nanoplasmonics for Amplified Surface Enhanced Raman Spectroscopy. ACS Nano.

[70] Kovalets N.P., Kozhina E.P., Razumovskaya I.V., Bedin S.A., Piryazev A.A., Grigoriev Y.V., Naumov A.V. (2022). Toward single-molecule surface-enhanced Raman scattering with novel type of metasurfaces synthesized by crack-stretching of metallized track-etched membranes. J. Chem. Phys..

[71] Nguyen T.V., Pham L.T., Khuyen B.X., Duong D.C., Nghiem L.H.T., Nguyen N.T., Vu D., Hoa D.Q., Lam V.D., Nguyen H.M. (2022). Effects of metallic underlayer on SERS performance of a metal film over nanosphere metasurface. J. Phys. D Appl. Phys..

[72] Mao P., Liu C., Chen Q., Han M., Maier S.A., Zhang S. (2020). Broadband SERS detection with disordered plasmonic hybrid aggregates. Nanoscale.

[73] Wang Y., Zhao C., Wang J., Luo X., Xie L., Zhan S., Kim J., Wang X., Liu X., Ying Y. (2021). Wearable plasmonic-metasurface sensor for noninvasive and universal molecular fingerprint detection on biointerfaces. Sci. Adv..

[74] Luo S., Mancini A., Berté R., Hoff B.H., Maier S.A., de Mello J.C. (2021). Massively Parallel Arrays of Size-Controlled Metallic Nanogaps with Gap-Widths Down to the Sub-3-nm Level. Adv. Mater..

[75] Bauman S.J., Darweesh A.A., Furr M., Magee M., Argyropoulos C., Herzog J.B. (2022). Tunable SERS Enhancement via Sub-nanometer Gap Metasurfaces. ACS Appl. Mater. Interfaces.

[76] Zhang W., Ai B., Gu P., Guan Y., Wang Z., Xiao Z., Zhang G. (2021). Plasmonic Chiral Metamaterials with Sub-10 nm Nanogaps. ACS Nano.

[77] Thareja V., Esfandyarpour M., Kik P.G., Brongersma M.L. (2019). Anisotropic Metasurfaces as Tunable SERS Substrates for 2D Materials. ACS Photonics.

[78] Jiang T., Goel P., Zhao H., Ma R., Zhu L., Liu X., Tang L. (2020). Internal Structure Tailoring in 3D Nanoplasmonic Metasurface for Surface-Enhanced Raman Spectroscopy. Part. Part. Syst. Charact..

[79] Leitis A., Tittl A., Liu M., Lee B.H., Gu M.B., Kivshar Y.S., Altug H. (2019). Angle-multiplexed all-dielectric metasurfaces for broadband molecular fingerprint retrieval. Sci. Adv..

[80] Tseng M.L., Jahani Y., Leitis A., Altug H. (2021). Dielectric Metasurfaces Enabling Advanced Optical Biosensors. ACS Photonics.

[81] Folland T.G., Lu G., Bruncz A., Nolen J.R., Tadjer M., Caldwell J.D. (2020). Vibrational Coupling to Epsilon-Near-Zero Waveguide Modes. ACS Photonics.

[82] Leitis A., Tseng M.L., John-Herpin A., Kivshar Y.S., Altug H. (2021). Wafer-Scale Functional Metasurfaces for Mid-Infrared Photonics and Biosensing. Adv. Mater..

[83] Autore M., Dolado I., Li P., Esteban R., Alfaro-Mozaz F.J., Atxabal A., Liu S., Edgar J.H., Velez S., Casanova F. (2021). Enhanced Light–Matter Interaction in ^10^B Monoisotopic Boron Nitride Infrared Nanoresonators. Adv. Optical Mater..

[84] Ulusoy Ghobadi T.G., Ghobadi A., Odabasi O., Karadas F., Ozbay E. (2022). Subwavelength Densely Packed Disordered Semiconductor Metasurface Units for Photoelectrochemical Hydrogen Generation. ACS Appl. Energy Mater..

[85] Hüttenhofer L., Golibrzuch M., Bienek O., Wendisch F.J., Lin R., Becherer M., Sharp I.D., Maier S.A., Cortes E. (2021). Metasurface Photoelectrodes for Enhanced Solar Fuel Generation. Adv. Energy Mater..

[86] Xiao Q., Kinnear C., Connell T.U., Kashif M.K., Easton C.D., Seeber A., Bourgeois L., Bonin G.O., Duffy N.W., Chesman A.S.R. (2021). Dual Photolytic Pathways in an Alloyed Plasmonic Near-Perfect Absorber: Implications for Photoelectrocatalysis. ACS Appl. Nano Mater..

[87] Deng S., Zhang B., Choo P., Smeets P.J.M., Odom T.W. (2021). Plasmonic Photoelectrocatalysis in Copper–Platinum Core–Shell Nanoparticle Lattices. Nano Lett..

[88] Loh J.Y.Y., Safari M., Mao C., Viasus C.J., Eleftheriades G.V., Ozin G.A., Kherani N.P. (2021). Near-Perfect Absorbing Copper Metamaterial for Solar Fuel Generation. Nano Lett..

[89] Capitolis J., Hamandi M., Hochedel M., El-Jallal S., Drouard E., Chevalier C., Leclercq J.-L., Penuelas J., Dursap T., Brottet S. (2022). Two-dimensional photonic metasurfaces for slow light-controlled photocatalysis. Nano Select.

[90] Hu H., Weber T., Bienek O., Wester A., Hüttenhofer L., Sharp I.D., Maier S.A., Tittl A., Cortes E. (2022). Catalytic Metasurfaces Empowered by Bound States in the Continuum. ACS Nano.

[91] Yu M.-J., Chang C.-L., Lan H.-Y., Chiao Z.-Y., Chen Y.-C., Lee H.W.H., Chang Y.-C., Chang S.-W., Tanaka T., Tung V. (2021). Plasmon-Enhanced Solar-Driven Hydrogen Evolution Using Titanium Nitride Metasurface Broadband Absorbers. ACS Photonics.

[92] Dutta A., Naldoni A., Malara F., Govorov A.O., Shalaev V.M., Boltasseva A. (2019). Gap-plasmon enhanced water splitting with ultrathin hematite films: The role of plasmonic-based light trapping and hot electrons. Faraday Discuss..

